# New Insights into Reelin-Mediated Signaling Pathways

**DOI:** 10.3389/fncel.2016.00122

**Published:** 2016-05-09

**Authors:** Gum Hwa Lee, Gabriella D’Arcangelo

**Affiliations:** ^1^College of Pharmacy, Chosun UniversityGwangju, South Korea; ^2^Department of Cell Biology and Neuroscience, Rutgers, The State University of New JerseyPiscataway, NJ, USA

**Keywords:** brain development, neuronal migration, dendrites, synaptogenesis, signal transduction

## Abstract

Reelin, a multifunctional extracellular protein that is important for mammalian brain development and function, is secreted by different cell types in the prenatal or postnatal brain. The spatiotemporal regulation of Reelin expression and distribution during development relates to its multifaceted function in the brain. Prenatally Reelin controls neuronal radial migration and proper positioning in cortical layers, whereas postnatally Reelin promotes neuronal maturation, synaptic formation and plasticity. The molecular mechanisms underlying the distinct biological functions of Reelin during and after brain development involve unique and overlapping signaling pathways that are activated following Reelin binding to its cell surface receptors. Distinct Reelin ligand isoforms, such as the full-length protein or fragments generated by proteolytic cleavage differentially affect the activity of downstream signaling pathways. In this review, we discuss recent advances in our understanding of the signaling transduction pathways activated by Reelin that regulate different aspects of brain development and function. A core signaling machinery, including ApoER2/VLDLR receptors, Src/Fyn kinases, and the adaptor protein Dab1, participates in all known aspects of Reelin biology. However, distinct downstream mechanisms, such as the Crk/Rap1 pathway and cell adhesion molecules, play crucial roles in the control of neuronal migration, whereas the PI3K/Akt/mTOR pathway appears to be more important for dendrite and spine development. Finally, the NMDA receptor (NMDAR) and an unidentified receptor contribute to the activation of the MEK/Erk1/2 pathway leading to the upregulation of genes involved in synaptic plasticity and learning. This knowledge may provide new insight into neurodevelopmental or neurodegenerative disorders that are associated with Reelin dysfunction.

## Introduction

Reelin is an extracellular glycoprotein that controls diverse aspects of mammalian brain development and function (D’Arcangelo, 2014). The most prominent activity of Reelin is the control of neuronal migration and cellular layer formation in the developing brain. This is evident from anatomical studies of *reeler* mutant mice that lack Reelin expression (Lambert de Rouvroit and Goffinet, 1998). These mutants exhibit a neurological phenotype characterized by ataxia and a typical “reeling” gate. Anatomically, their brains exhibit widespread neuronal lamination defects due to the failure of radially-migrating neurons to reach their destination in the developing forebrain, and cerebellar hypoplasia, which is likely due to the failure of Purkinje cells to form a cellular layer (Goffinet, 1983; Miyata et al., 1997). Similar phenotypes are observed in human patients carrying *REELIN* homozygous mutations, resulting in lissencephaly with cerebellar hypoplasia (Hong et al., 2000).

In addition to controlling neuronal migration in the prenatal brain, Reelin plays important roles in the postnatal and adult brain, promoting the maturation of dendrites, synaptogenesis, synaptic transmission and plasticity, thus modulating the formation and function of synaptic circuits. This view is supported not only by animal studies involving heterozygous *reeler* mice, which model some behavioral dysfunction similar to schizophrenia (Costa et al., 2002), but also by recent human genetic studies identifying heterozygous *REELIN* mutations in lateral temporal epilepsy (Dazzo et al., 2015), and pointing to *REELIN* as a risk factor in autism (De Rubeis et al., 2014). Furthermore, accumulating evidence that Reelin signaling antagonizes the toxic effects of β-amyloid at the synapse, underscores the potential relevance of this “developmental” factor for neurodegenerative disorders (Durakoglugil et al., 2009; Krstic et al., 2012; Pujadas et al., 2014).

To foster a better understanding of the mechanisms of development and disease, in this review we focus on recent advances in our knowledge of the signaling transduction pathways that regulate the different biological activities of Reelin in the brain.

## Reelin Expression and Cleavage

The spatiotemporal regulation of Reelin expression underlies its multifaceted roles in brain development. During the embryonic development of forebrain structures Cajal-Retzius cells secrete high levels of Reelin in the marginal zone, thus regulating neuronal migration and cellular layer formation (D’Arcangelo et al., 1995; Ogawa et al., 1995). These cells begin to die shortly after birth and disappear from the neocortex once neuronal migration is completed. In the hippocampus, however, residual Cajal-Retzius continue to secrete Reelin at early postnatal days, affecting aspects of development such as axonal or dendrite branching and maturation (Del Río et al., 1997; Niu et al., 2004; Kupferman et al., 2014). As postnatal development continues, the expression of Reelin becomes predominantly localized to a subset of GABAergic interneurons that are positioned throughout cortical and hippocampal cell layers (Alcántara et al., 1998; Pesold et al., 1998). Albeit at reduced levels, these interneurons continue to express Reelin in the juvenile and adult forebrain. The significance of this late postnatal and adult pattern of expression is likely related to the modulation of synaptic activity and plasticity (Weeber et al., 2002; Beffert et al., 2005; Pujadas et al., 2010; Trotter et al., 2013).

**Figure 1:**
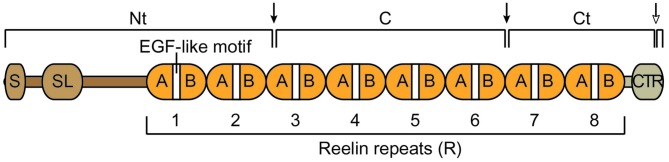
**Schematic structure of the Reelin protein and its cleavage fragments.** Reelin contains a signal peptide (S), an F-spondin-like domain (SL), eight consecutive Reelin repeats (R) each harboring an epidermal growth factor (EGF)-like motif that separates two subdomains (A and B), and a positively charged carboxy-terminal region (CTR). The full-length protein is cleaved by extracellular metalloproteases at specific sites (arrows), an N-terminal (Nt) site within R3 and a C-terminal site between R6 and R7. These two cleavage events generate three large fragments, an N-terminal (Nt), a central (C) and a C terminal (Ct) fragment. An additional cleavage event (empty head arrow) occurs within the CTR (WC) and generates a small carboxy-terminal peptide.

The mouse full-length Reelin protein is approximately 385 kDa and is 95.2% identical to the human protein (D’Arcangelo et al., 1995). The main body of the protein is composed of eight unique repeats (R), each centered around an epidermal growth factor (EGF)-like cysteine pattern that is typical of extracellular proteins (Figure 1). At the N terminus there is a signal peptide and a small region of similarity with F-spondin, whereas at the C terminus there is a small carboxy-terminal region (CTR) that is positively charged. The presence of the signal peptide indicated that Reelin is an extracellular protein. Indeed, it is readily detected in the culture medium of Reelin-expressing cells (D’Arcangelo et al., 1997). Secretion is essential for function, and mutations that interfere with secretion cause a *reeler* phenotype identical to that resulting from null mutations (D’Arcangelo et al., 1997; de Bergeyck et al., 1997). After secretion, full-length Reelin is cleaved by metalloproteases at two specific sites, generating three large fragments, an N-terminal (Nt = N-R2), a central (C = R3-R6), and a C-terminal (Ct = R6-CTR) fragment (Figure 1). The C fragment alone is sufficient to activate intracellular signaling and to induce layer formation in cortical slice cultures (Jossin et al., 2004; Yasui et al., 2007). However, the full-length protein is more potent than the C fragment, presumably due to the presence of the Nt region, which promotes aggregation, and the CTR, which promotes proper folding (Utsunomiya-Tate et al., 2000; Kubo et al., 2002; Nakano et al., 2007; Kohno et al., 2015). Recent studies identified the cleavage sites that produce the three major Reelin fragments (Koie et al., 2014; Sato et al., 2016) and demonstrated that the Nt cleavage affects the duration and the range of Reelin signaling activity in the developing cortex (Koie et al., 2014). Further studies are needed to identify proteases that carry out these processing events *in vivo*. In addition, recent studies further identified another cleavage site within the CTR (WC). Cleavage at this site releases a six amino acid carboxy-terminal peptide, reducing signaling activity and hindering dendrite development in the postnatal neocortex (Kohno et al., 2015).

Taken together, the evidence so far indicates that Reelin processing downregulates the activity of the full-length protein; however cleavage events also produce diffusible fragments that potentially stimulate signaling activity away from the site of secretion (Jossin et al., 2007).

## Reelin Receptors

The best-characterized Reelin receptors are the apolipoprotein E receptor 2 (ApoER2, also called LRP8) and the very low-density lipoprotein receptor (VLDLR). These proteins belong to the low-density lipoprotein receptor (LDLR) family. They have partial functional redundancy and play an essential role in Reelin-mediated neuronal migration based on the observation that double knockout mice display a *reeler*-like phenotype (Trommsdorff et al., 1999). ApoER2 and VLDLR bind Reelin with high affinity and internalize the ligand in endocytic vesicles, leading to the activation of downstream signaling molecules (D’Arcangelo et al., 1999; Hiesberger et al., 1999; Strasser et al., 2004; Yasui et al., 2010). After the signal is transduced, some receptor molecules recycle to the membrane whereas others are targeted for lysosomal degradation (Hong et al., 2010). A Reelin domain contained within the C fragment and including the Lys2467 residue is essential for ApoER2/VLDLR interaction, signal transduction and cortical layer formation (Jossin et al., 2004; Yasui et al., 2007). Despite functional overlap, ApoER2 and VLDLR play distinct roles in neuronal migration due, in part, to their different expression pattern. In the developing neocortex VLDLR is expressed almost exclusively in apical processes of migrating neurons at the top of the cortical plate where it mediates a mode of migration known as somal or terminal translocation, whereas ApoER2 is also expressed in the intermediate zone where it likely promotes the transition from multipolar to bipolar morphology and early stages of radial migration (Hirota et al., 2015). Other reported differences between the two receptors include their ability to internalize Reelin at different rate and in distinct lipid compartments, thus likely differentially affecting signal transduction machineries (Duit et al., 2010).

Other transmembrane proteins that have been proposed to function as Reelin receptors include β1-containing integrins, which were first reported to bind Reelin *in vitro* (Dulabon et al., 2000). However, genetic knock out studies later demonstrated that β1 integrins are required for radial glia scaffold formation rather than for neuronal migration *per se* (Belvindrah et al., 2007). Even though their function is not essential, possibly due to redundancy with other cell adhesion molecules, *in utero* electroporation studies suggest that β1 integrins contribute to corticogenesis as downstream effectors. Reelin signaling was shown to alter integrin-dependent cell adhesion by downregulating α3 integrin levels in the cortical plate (Sanada et al., 2004), and by activating integrin α5β1, thus promoting the anchoring of leading processes to the fibronectin-rich marginal zone (Sekine et al., 2012). It should be noted that in this model integrins do not bind Reelin directly and therefore do not function as receptors. Recently, another study suggested a direct interaction between Reelin and EphB tyrosine kinase receptors. The Nt region of Reelin was reported to bind EphB and activate forward signaling in neurons (Bouché et al., 2013). However, EphB-deficient mice display only a very mild migration phenotype, suggesting that they do not play a major role during prenatal brain development. Their involvement in postnatal functions of Reelin remains to be elucidated.

Taken together, genetic and biochemical data so far support the notion that ApoER2 and VLDLR are the major Reelin receptors in the developing brain.

## Reelin Signal Transduction in the Control of Neuronal Migration

**Figure 2:**
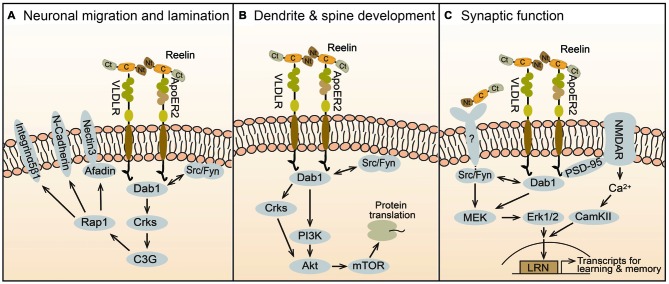
**Reelin signaling mechanisms in brain development and function.** Reelin is secreted as a full-length protein that contains three large cleavable domains, an Nt, a C, and a Ct domain. The central domain binds to ApoER2 and VLDLR receptors, which internalize the ligand and transduce the Reelin signal by activating Src/Fyn kinases that phosphorylate the adaptor protein Dab1. Downstream of this canonical pathway, distinct signaling cascades regulate specific biologic activities at different times during brain development. **(A)** Prenatally, Reelin controls neuronal migration and cortical layer formation through the Crk/C3G/Rap1 pathway. This signaling cascade regulates the function of cell adhesion molecules, including nectin3, N-Cadherin, and Integrin α5β1, which facilitate somal translocation and cellular layer formation. **(B)** During early postnatal development, the Crk adaptor proteins and the PI3K-Akt-mTOR pathway contribute to Reelin activity by promoting protein translation, dendrite outgrowth and spine development. **(C)** In the late postnatal and adult brain Reelin affects synaptic function and plasticity. This activity is mediated in part by ApoER2, which interacts with the NMDAR through PSD-95, causing Ca^2+^ influx and the activation of CamKII. An unknown receptor also mediates the activation of the MEK-Erk1/2 pathway by Src/Fyn kinases. Together these signaling pathways promote synaptic activity and plasticity through the induction of immediate-early genes involved in learning and memory such as those containing LRN enhancers.

Disabled-1 (Dab1) is an intracellular adaptor protein that is essential for Reelin signal transduction. This protein binds the cytoplasmic tail of lipoprotein receptors, including ApoER2 and VLDLR (Trommsdorff et al., 1999) and upon Reelin binding, becomes phosphorylated on tyrosine residues by Src-family kinases (SFKs) Fyn and Src (Howell et al., 1999a; Figure 2A). These kinases are themselves upregulated in a Dab1-dependent way via a positive feedback mechanism (Arnaud et al., 2003; Bock and Herz, 2003). Dab1 phosphorylation is required for neuronal migration, as demonstrated by the observation that phospho-mutant Dab1 mice (Howell et al., 2000), double *Fyn/Src* knockout mice (Kuo et al., 2005), as well as spontaneous or genetically engineered *Dab1* knockout mice (Howell et al., 1997; Sheldon et al., 1997; Ware et al., 1997; Yoneshima et al., 1997; Kojima et al., 2000) all show similar *reeler*-like phenotypes. Dab1 signaling is rapidly downregulated by a mechanism that involves the ubiquitination of phospho-Dab1 by the E3 ubiquitin ligase component Cullin 5, and its degradation by the proteasome system (Feng et al., 2007).

Genetic studies demonstrated that Dab1, and thus Reelin signaling, is specifically required for a specific mode of radial migration termed somal or terminal translocation, but not for glial-guided locomotion (Franco et al., 2011). The molecular mechanism of translocation involves the recruitment of Crk adaptor proteins, which bind phospho-Dab1 and cause the activation of the GTP exchange factor (GEF) C3G, and the subsequent activation of the Rap1 GTPase (Franco et al., 2011; Jossin and Cooper, 2011; Figure 2A). Consistently, double *Crk*/*CrkL* mutant mice display a *reeler*-like cortical phenotype (Park and Curran, 2008). The Crk/C3G/Rap1 pathway ultimately promotes the interaction between migrating neurons and Reelin-producing Cajal-Retzius cells through adhesion molecules such as nectins 1/3 and N-Cadherin, enabling neuronal translocation and inside-out layer formation (Gil-Sanz et al., 2013; Figure 2A). Given the enrichment of ApoER2 and VLDLR in the apical processes of migrating neurons near the marginal zone, both these receptors are likely to mediate the signal transduction that promotes translocation (Hirota et al., 2015). In addition, Reelin-Dab1 signaling through Rap1 and N-Cadherin affects the orientation of migrating neurons undergoing the transition from multipolar to bipolar morphology in the intermediate zone, before initiating radial migration into the cortical plate (Jossin and Cooper, 2011). This migration step may be mediated preferentially by ApoER2, since this is the only receptor that is expressed in the intermediate zone (Hirota et al., 2015).

In addition to Crks and Rap1, biochemical studies identified several molecules that may be involved in Reelin-dependent neuronal migration. These include proteins that regulate cytoskeletal dynamics and cell motility, such as Lis1, Nckβ and N-WASP (Assadi et al., 2003; Pramatarova et al., 2003; Suetsugu et al., 2004), and proteins that downregulate Rap1 due to their GTPase activating protein (GAP) activity. Among Dab1-interacting proteins Lis1, the product of the *PAFAH1b1* gene that is responsible for human lissencephaly type I, may be particularly relevant to cortical development. Lis1 binding to phospho-Dab1 is Reelin-dependent, and genetic interaction between *Dab1* and *PAFAH1b1* demonstrates a functional relationship between these proteins (Assadi et al., 2003). Furthermore, Lis1-interacting *PAFAH1b* alpha subunits bind specifically to VLDLR, potentially promoting the interaction between Lis1 and Dab1 downstream of this receptor (Zhang et al., 2007). Lis1 then affects cytoskeletal dynamics necessary for radial migration through the dynein motor complex (Wynshaw-Boris and Gambello, 2001). Additionally, Dab2IP, a Dab1-binding protein that functions as a Rap GAP, as well as Rap1GAP, were shown to affect neuronal migration in the neocortex (Franco et al., 2011; Jossin and Cooper, 2011; Lee et al., 2012; Qiao et al., 2013). Even though a direct involvement of Rap GAPs in Reelin signaling has not been established, it is likely that this class of proteins regulates Rap1 activity, balancing the GEF activity of C3G and thus enabling proper neuronal orientation and migration through the cortical plate.

## Reelin Signal Transduction in the Control of Dendrite and Spine Development

Dendrite outgrowth is disrupted in homozygous *reeler* mice. Dendritic defects are also apparent in immature hippocampal or cortical cultures isolated from mutant mice, but not in mature cultures (Niu et al., 2004; Jossin and Goffinet, 2007; MacLaurin et al., 2007)*.* Since Reelin treatment rescued these defects, these *in vitro* studies first demonstrated that Reelin directly promotes dendrite development. Following studies further demonstrated that Reelin enables initial dendritic outgrowth by promoting the extension of the Golgi apparatus into apical dendrites (Matsuki et al., 2010), and then orienting and stabilizing the leading processes in the marginal zone (Chai et al., 2015; Kohno et al., 2015; O’Dell et al., 2015). The signal transduction machinery that mediates the activity of Reelin on dendrite development involves the canonical pathway that also controls neuronal migration, including ApoER2/VLDLR, Dab1, SFKs and Crks (Niu et al., 2004; Park and Curran, 2008). Downstream of Dab1, the signaling mechanism that affects dendrite development likely involves the Phosphoinositide 3-kinase (PI3K) and Akt (Figure 2B). Earlier studies demonstrated that Reelin activates PI3K and Akt *in vitro* in a manner that is dependent on SFK activity and Dab1 phosphorylation (Beffert et al., 2002; Bock et al., 2003). PI3K may be activated through direct interaction between the regulatory subunit p85α and Dab1 (Bock et al., 2003). Akt is likely activated, at least in part, by the classic PI3K/PDK cascade, however, *in vivo* studies demonstrated that the Crk adaptor proteins are required for Reelin-induced Akt phosphorylation, placing the kinase functionally downstream of these adaptors (Park and Curran, 2008). Downstream of Akt, mTOR and further downstream proteins such as p70S6K and ribosomal protein S6 are robustly induced by Reelin treatment in neuronal cultures and likely contribute to dendrite growth (Jossin and Goffinet, 2007; Ventruti et al., 2011; Figure 2B).

Other molecules that have been implicated in Reelin-dependent dendrite outgrowth include the amyloid precursor protein (APP; Hoe et al., 2009), which binds Dab1 via its cytoplasmic tail (Homayouni et al., 1999; Howell et al., 1999b), and the Cdc42/Rac1 guanine nucleotide exchange factor αPIX, which affects dendritic Golgi translocation (Meseke et al., 2013). In addition to outgrowth, dendrite compartmentalization is an important aspect of maturation that is affected by Reelin. In the hippocampus, distal apical dendrites of pyramidal neurons express specific ion channels. Recent studies demonstrated that Dab1/SFK signaling is required for the molecular identity of this dendritic compartment, which regulates the processing of information in hippocampal circuits (Kupferman et al., 2014). Reelin signaling also promotes dendritic spine formation and growth in the cortex and hippocampus of juvenile mice (Niu et al., 2008; Pujadas et al., 2010; Iafrati et al., 2014). The signaling mechanism that underlies this function involves the canonical pathway and possibly additional signaling molecules such as RasGRF1/CamMKII (DiBattista et al., 2015; Kim et al., 2015). Finally, the molecular composition of the dendritic spines is affected by Reelin. Specifically, Reelin promotes the maturation of spines by regulating the NMDA receptor (NMDAR) subunit composition via an unidentified mechanism (Groc et al., 2007; Ventruti et al., 2011).

## Reelin Signaling and the Modulation of Synaptic Function

Heterozygous *reeler* mice exhibit altered hippocampal synaptic plasticity and multiple behavioral abnormalities, such as defects in executive function and contextual fear conditioning learning (Brigman et al., 2006; Krueger et al., 2006; Qiu et al., 2006). Early culture studies demonstrated that Reelin potently enhances hippocampal long-term potentiation (LTP), a cellular mechanism underlying learning and memory, and this effect is dependent on the presence of both, VLDLR and ApoER2 (Weeber et al., 2002). A specific splicing variant of ApoER2 was required for Reelin-induced LTP enhancement and memory formation *in vivo* (Beffert et al., 2005). Mechanistically, it was shown that this ApoER2 variant interacts with the NMDAR through PSD-95, and this complex mediates Reelin–induced Ca^++^ influx through the NMDAR (Beffert et al., 2005; Chen et al., 2005; Figure 2C). Genetic studies later demonstrated that Dab1 is also required for Reelin-induced enhancement of hippocampal LTP and for hippocampal-dependent behavioral tasks (Trotter et al., 2013). This study also demonstrated that postnatal Dab1 loss affects basal and plasticity-induced Erk1/2 signaling, suggesting a cross-talk with canonical Reelin signaling. Indeed, Reelin was shown to induce Erk1/2 signaling in a SFK-dependent manner in cultured neurons (Lee et al., 2014). Surprisingly, however, Reelin-induced Erk1/2 phosphorylation did not require the activity of ApoER2 and VLDLR, and it was only partially dependent on Dab1, suggesting the involvement of an unidentified receptor triggering a non-canonical pathway (Figure 2C). Erk1/2 activation leads to the expression of synaptic immediate-early genes (IEGs), and thus potentially affects synaptic function (Lee et al., 2014). Others further showed that Reelin induces IEGs expression via a novel enhancer element named LRN (LRP8-Reelin-Neuronal), and that these events affect associative learning. In this model, interaction between the ApoER2 (LRP8) and the NMDAR triggers Ca^++^ influx, Erk1/2 signaling and CREB-dependent IEGs transcription (Telese et al., 2015). In addition, they reported that proteolytical cleavage of ApoER2 by γ-secretase is a crucial component of the synapse-to-nuclear signaling triggered by Reelin. Interestingly, Notch1, another γ-secretase substrate, was also recently shown to contribute to Reelin-mediated synaptic potentiation by interacting with ApoER2 and NMDAR, and stimulating Erk1/2 activity and CREB-dependent transcription (Brai et al., 2015).

In addition to its well-documented postsynaptic effects, Reelin also acts presynaptically, causing a rapid enhancement of spontaneous neurotransmitter release. This effect is due to the mobilization of VAMP7-containing synaptic vesicles, and requires canonical ApoER2/VLDLR receptors, PI3K and Ca^++^ signaling (Hellwig et al., 2011; Bal et al., 2013). Despite robust pre- and postsynaptic effects, acute deletion of the *Reelin* gene in adult mice does not result in impaired synaptic plasticity. However, it renders the adult brain strikingly sensitive to amyloid-induced synaptic suppression, leading to profound learning disabilities (Lane-Donovan et al., 2015). Although specific molecular and physiological mechanisms remain to be further elucidated, these findings indicate that Reelin has the potential to modulate synaptic activity and thus affect memory formation in the adult and aging brain.

## Author Contributions

GHL wrote the first draft of the manuscript and made the figures. GD wrote and revised the manuscript.

## Conflict of Interest Statement

The authors declare that the research was conducted in the absence of any commercial or financial relationships that could be construed as a potential conflict of interest.
